# Evaluation of acute flaccid paralysis surveillance system in Kebbi State, Nigeria between 2013-2018

**DOI:** 10.11604/pamj.2025.50.84.46398

**Published:** 2025-03-25

**Authors:** Abdulmumin Hashim Bala, Yahaya Mohammed, Olajide Adewale Owolodun, Lukman Surajudeen, Ismail Abdullateef Raji, Charles Rahab Amaza

**Affiliations:** 1 African Field Epidemiology Network, Abuja, Nigeria,; 2 College of Health Sciences, Usmanu Danfodiyo University Sokoto, Sokoto, Nigeria,; 3 National Veterinary Research Institute, Vom, Jos, Plateau State, Nigeria,; 4 World Health Organization, Birnin Kebbi, Kebbi State, Nigeria,; 5 Africa Center for Disease Control and Prevention, Addis Ababa, Ethiopia

**Keywords:** Acute flaccid paralysis, evaluation, disease notification, surveillance systems, poliomyelitis, public health surveillance, epidemiological monitoring, vaccination coverage, Kebbi State, Nigeria

## Abstract

**Introduction:**

since 1988, polio incidence has declined by over 99% globally, from more than 350,000 annual cases in over 125 endemic countries to transmission now limited to Pakistan and Afghanistan. Africa has not reported wild poliovirus since 2016. An effective Acute Flaccid Paralysis (AFP) surveillance system is crucial for detecting and interrupting polio transmission. This evaluation assessed the Acute Flaccid Paralysis (AFP) surveillance system in Kebbi State, Nigeria, to identify operational gaps.

**Methods:**

using updated Center for Disease Control and Prevention (CDC) guidelines and the World Health Organization (WHO) performance standards, the study reviewed AFP surveillance data from 2013-2018, conducted stakeholder interviews using adapted questionnaires and key informant interviews, and analyzed data using means, frequencies, and proportions.

**Results:**

among the 49 respondents, 98% reported that case definitions and investigation forms were easy to use, while 97% found data tools adaptable to changes. All surveillance officers understood AFP case definitions and expressed willingness to sustain the system. Key performance indicators, including non-polio AFP rates (24.6-55.2), stool adequacy (95-99.7%), timeliness, and reporting completeness all consistently met WHO standards.

**Conclusion:**

the evaluation concluded that the AFP surveillance system in Kebbi State, Nigeria, is useful, simple, flexible, acceptable, sensitive, representative, timely, and stable, although donor dependency remains a concern. A significant gap was the lack of data on 60-day follow-ups and laboratory feedback. Addressing these issues is important to further strengthen the system.

## Introduction

Poliomyelitis, caused by poliovirus serotypes 1, 2, and 3, is a highly contagious viral disease that can result in severe outcomes [1]. The majority of infections are asymptomatic, but a small percentage can lead to muscle weakness and flaccid paralysis, primarily affecting the legs but sometimes the head, neck, or diaphragm. Severe cases can cause respiratory complications and death, particularly among children (2-5%) and adults (15-30%) with paralysis [2]. Acute Flaccid Paralysis (AFP) is a condition characterized by sudden weakness in one or more limbs. The syndrome may result from different causes such as poliomyelitis, Guillain-Barré Syndrome (GBS) and non-polio enteroviruses [3]. While there are similarities in the clinical manifestations of AFP and GBS, the two conditions are different. GBS is a rare autoimmune disease that commonly occurs after a prior infection with the body's immune system turning against the nervous system, leading to a rapid onset of muscle weakness with often absent reflexes [4]. On the other hand, AFP due to poliomyelitis typically presents with a more acute onset of asymmetric paralysis and diminished or absent reflexes in the affected limbs. Differentiating between AFP and GBS is necessary to achieve an accurate diagnosis, effective management and prompt notification [3,4]. Vaccination remains the cornerstone of polio prevention, with two vaccines available: the oral polio vaccine (OPV) and the inactivated polio vaccine (IPV) [5]. OPV is widely used due to its ease of administration, ability to provide intestinal immunity, and low cost, though it carries a rare risk of vaccine-associated paralytic poliomyelitis (VAPP) [5]. The global polio eradication initiative has achieved significant progress, with wild poliovirus types 2 and 3 eradicated. Only type 1 remains, confined to Pakistan and Afghanistan [6]. In 2020, the WHO African Region was declared wild poliovirus-free after four years without any reported cases, marking an important milestone toward global eradication [7].

Acute Flaccid Paralysis (AFP) surveillance plays an important role in detecting and interrupting poliovirus transmission. It involves monitoring children under 15 years with sudden limb weakness, distinguishing polio from other conditions like Guillain-Barré Syndrome [8]. Effective AFP surveillance is essential for verifying the absence of wild poliovirus in polio-free regions [9]. Nigeria's AFP surveillance system has been instrumental in identifying high-transmission areas and guiding interventions. The system is supported by WHO, Rotary International, African Field Epidemiology Network (AFENET), UNICEF, Agency for International Development (USAID), and U.S. Centers for Disease Control and Prevention (U.S. CDC).

The purpose of evaluating public health surveillance systems is to ensure that problems of public health importance are being monitored efficiently and effectively [9]. Furthermore, evaluation of a surveillance system helps to determine if a system is meeting the set objectives and whether the attributes are efficient to achieve these objectives [9]. We described the operations of the Kebbi State AFP surveillance system to assess its key attributes and performance of the system in line with its set objectives.

## Methods

**Study area:** Kebbi is a state in the north western part of Nigeria. The 2019 projected population of Kebbi State was 4,671,594 while the under-five population was 2,223,679 [10]. It has 21 Local Government Areas (LGAs), 225 political wards, and 122 districts spread over four emirate councils namely Argungu, Gwandu, Yauri, and Zuru. Seventy percent of the people live in rural communities, where the predominant economic activities are fishing, farming, and trading. There are 22 general hospitals, 225 primary health care centres, a Federal Medical centre, and a Specialist Hospital. In total, there are 203 focal sites for AFP which include all the secondary and tertiary public health facilities and all the facilities offering routine immunization (RI) services offer free RI services.

**Study design:** we conducted a descriptive, cross-sectional study.

**Overview of AFP surveillance in Kebbi State:** the AFP surveillance in Kebbi State is collated at the State level after receiving information from the various local government areas (LGA). At the LGA level, the Disease Surveillance and Notification Officers (DSNOs), health facility focal persons and other staff working with donor and implementing organizations like the WHO conduct active case searches and detect, and report suspected cases to the LGA DSNOs. Passive surveillance is also conducted via community informants (such as traditional healers and community health workers). Thereafter, the DSNO at the LGA level assign EPID numbers, completes Essential Case Forms (ECFs), collects samples, and verifies cases. The DSNOs submit weekly reports, including zero reporting, to the State DSNOs and this is what forms the state report. These activities are funded by Donors with little or no government support. Private facilities do not report at all. This study examines all aspects of this reporting system.

**Study population, study tools and data management (quantitative study):** a structured self-administered questionnaire, adapted from previous similar studies and adapted for this study was used to obtain information from respondents (these are some of the respondents; State Epidemiologist, State Disease Surveillance and Notification Officer (DSNO), Assistant State DSNO, Local Government Area (LGA) DSNOs/Assistant Disease Surveillance and Notification Officer (ADSNOs). This is to assess their views on some of the surveillance system attributes (such as the simplicity, flexibility, representativeness, stability, usefulness, and acceptability of the system). Another set of surveillance officers that were interviewed were the 'Community informants'. They are the traditional healers, herbalists, patent medicine vendors (PMVs) and bone setters, who have been trained on case definitions and thought to report any suspected cases. Table 1 shows the AFP key attributes, surveillance indicators and source of information.

**Table 1:** acute flaccid paralysis key indicator surveillance data

Surveillance Attributes	Key indicators	Source of information
**Data quality**	Timeliness and completeness of reporting	≥90%; all reports should be at least 90% complete and at least 90% should reach the State on time (WHO target)
**Sensitivity**	Annualized NP-AFPR	NP-AFPR should be ≥ 1/100,000/year of under 15
	Stool adequacy	Stool adequacy: > 80% of stool samples must be adequate (WHO target)
**Simplicity**	Simplicity of case definition	From staff interviews
	Ease of filling the CIF	
**Flexibility**	How easily the system integrates into IDSR	From staff interviews
	How easily the system can accommodate changes	
**Acceptability**	The willingness of the surveillance officers/stakeholders to continue with the surveillance as well as the acceptance by the communities involved	From staff interviews
**Stability**	Presence of dedicated staff to carry out AFP surveillance, presence of structure as well as funding of the system	From staff interview
**Representativeness**	The ability of the system to be carried out in all parts of the State, touching all communities (urban and rural), both males and females	From the data review
**Timeliness**	Timeliness of case investigation of reported AFP cases	All reported cases should be investigated within 24-48 hours
	Timeliness of stool sample arriving the laboratory	All stool samples should arrive the laboratory within 72 hours of collection (WHO criteria)

**Study population, study tools and data management (qualitative study):** a Key Informant Interview (KII) was conducted among some Key informants which include the SE, State DSNO, and some randomly selected three medical officers from the State general hospitals as well as the WHO surveillance focal person in the State. These were to obtain more information regarding acceptability, stability, representativeness, and challenges. The Key Informants were the top surveillance officers in the State who received all the data from the LGA DSNOs on a weekly and monthly basis. They analyse the data (State Epidemiologist and State DSNO) and present it to the State Commissioner of Health and the Director of Public Health. They also provide feedback to Health facility heads (medical officers who are also an integral part of the Surveillance System). They all participate in monthly review meetings for monitoring and evaluation of the Surveillance system. The key informant interview guide was adapted from the WHO framework guide on surveillance systems evaluation [11]. It was used to gather information from relevant stakeholders (State epidemiologists, medical officers, and monitoring and evaluation officers), and on stability representativeness, funding, and other challenges the system encountered. Those selected for KII include: the Kebbi State epidemiologists, Deputy State DSNO, Chief Medical Officers in General Hospital Zauro and General Hospital Zuru.

**Study tools and data management (review of documents):** we also used the WHO standard guidelines for AFP performance standards to assess some attributes of the AFP surveillance system evaluation, such as the sensitivity, timeliness, and data quality of the system. A retrospective record review of the AFP surveillance data from 2013-2018 was carried out. We used the data to determine the sensitivity, timeliness, and representativeness of the system. We assessed data quality by determining the timeliness and completeness of reporting. Completeness was assessed by estimating the proportion of complete weekly reports that got to the State by the close of work (4.00 pm) on Tuesday of the reporting week. Representativeness was calculated by determining the proportion of all the health facilities that submitted a report.

**Data analysis:** we cleaned, coded, and analysed the quantitative data we obtained from the self-administered questionnaires using IBM SPSS version 25. However, we used Microsoft Excel to co-conduct a secondary data analysis of the AFP surveillance data from January 2013 to December 2018. AFP notified cases aged <15 years old between January 2013 and December 2018 were analysed. The analysis was performed using Microsoft Excel version 2016. Evidence regarding performances was assessed using system attributes according to the CDCs updated guidelines to describe the attributes of the surveillance system. For the qualitative data obtained, we conducted a thematic analysis. For the thematic analysis, the following steps were followed:

***Step 1:*** familiarization; this was done by a thorough overview of all the data we collected.

***Step 2:*** coding the information obtained e.g. how simple is the case definition of AFP? Some responses were it's simple, or it is very simple, or it's very easy to understand, or it is very easy to remember etc. all these were coded as “easy to understand”.

***Step 3:*** generating themes: such as easy to understand, easy to accommodate changes, case definitions are easy to remember, etc.

**Ethical consideration:** written approval was first obtained from the Public Health Department of the Kebbi State Ministry of Health (Document No: MOH/STA/PER/6007/205751), followed by ethical clearance from the Kebbi State Ministry of Health Ethics Committee. All participants, who were key stakeholders, were interviewed privately, and the confidentiality of their responses was ensured. Written informed consent was also obtained from each participant, as documented in the questionnaire.

## Results

Four thousand five hundred and thirteen cases (4513) of AFP were reported between January 2013 to December 2018, from all LGAs. Fakai and Ngaski LGAs reported 362 (8%) cases, while Bagudo reported 45 (1%). Non-polio AFP rate (NP-AFPR) for Ngaski and Fakai LGAs were 71.5/100,000 and 73.8/100,000 respectively, while Wasagu/Danko had NP-AFP rate of 20.1/100,000.

**Data quality:** the data is also valid because the surveillance system aims to detect AFP cases and the system has been consistently and effectively reporting AFP cases throughout the review period. Ninety-seven percent of data fields used were complete.

**Figure 1:**
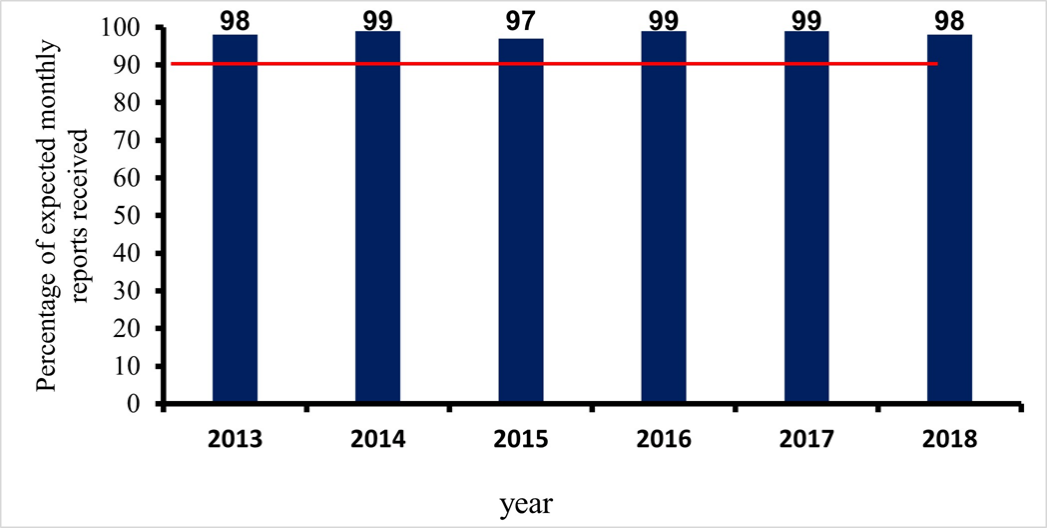
percentage of all monthly reports received

**Timeliness and completeness of reporting:** in the years under review, the timeliness of monthly reporting was consistently above 90%. In Figure 1, the least attained percentage by the surveillance system was 98% (in 2013 and 2018). The WHO cut off mark is 90%.

**Simplicity:** all the respondents knew that polio is a disease caused by a virus. 47 (97.6%) responded that the AFP case definition is easy to understand. The majority of the respondents, 45 (97%), felt the case investigation form CIF is easy to fill, however, 33 (70%) believed there is a need for regular training and retraining.

**Flexibility:** the AFP surveillance is well integrated into the Integrated Disease Surveillance and Response (IDSR) system. The AFP surveillance system uses the minimum data collection recommended by the WHO which are few. It can easily accommodate new specific variables as needed. However, 50% of the respondents think that the data collection forms can provide changes in the surveillance system process.

**Acceptability:** the system was generally acceptable by the surveillance officers. All of them were willing to continue to participate in the AFP surveillance in the state. Interviews with focal persons in some healthcare facilities showed they are willing to continue with AFP surveillance despite their busy schedules. Community informants were always willing to participate in the reporting of suspected cases. Nonetheless, some private healthcare facilities were not fully reporting. However, despite their willingness to continue with the current system, 62% of the respondents said they have made contributions or suggestions on areas where they think the system can be improved. Among the suggestions were, the surveillance officers should be supported especially with means of transportation to improve active case search, to increase the number of focal sites, or to completely change the reporting system to electronic format. The majority, 74% of the respondents said their suggestions were considered and some were even implemented (e.g. they were supported with motorcycles, android phones, and the number of focal sites were increased.

**Stability:** there are dedicated surveillance officers from the State Ministry of Health (i.e. The State Epidemiologist, State DSNO, and his Deputy), from the Local Government Authorities (LGAs) (i.e. LGA DSNOs and their Deputies), Health Facility surveillance focal persons, Community Informants, etc. The State is also supported by The WHO Cluster Consultants, WHO LGA facilitators, Field Volunteers, etc. therefore, the AFP surveillance system in Kebbi State is stable. However, the system is donor driven. The WHO provides all the logistic support, laboratory reagents and consumables, sample collection bottles, and transportation of samples to the reference laboratories. Therefore, the system will be unstable the moment the donors withdraw their support.

**Figure 2:**
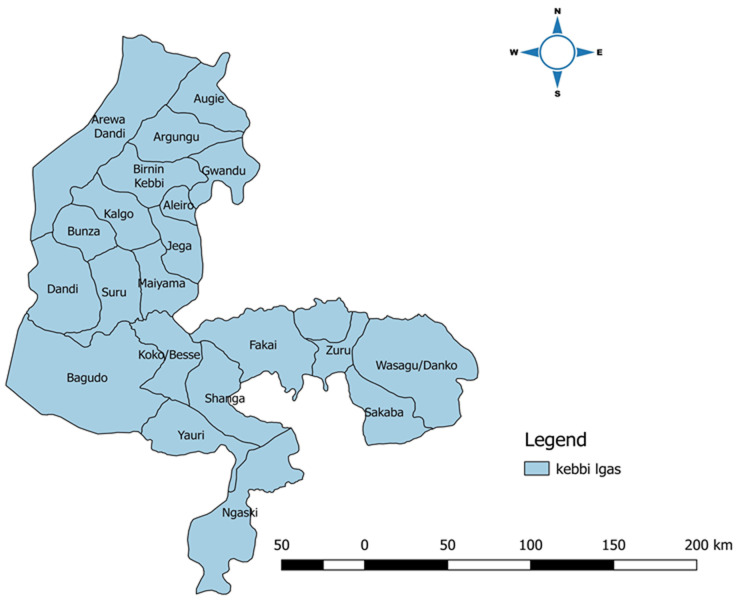
map of Kebbi State showing all the local government areas where acute flaccid paralysis surveillance is ongoing

**Representativeness:** the AFP surveillance system in the State is representative because the surveillance data analysed showed that the system is ongoing in all the LGAs in the State. Both males and females were represented in the data. Though there were more males (53%) than females (47%). It cuts across individuals from different ethnic backgrounds within the State, suspected cases cuts across individuals from different socioeconomic backgrounds, and all from both urban and rural settlements. The data is also obtained from both public and private health facilities. Figure 2 is the map of Kebbi State, showing all the Local Governments involved in AFP surveillance. The map depicts that all the 21 LGAs were reporting throughout the review.

**Figure 3:**
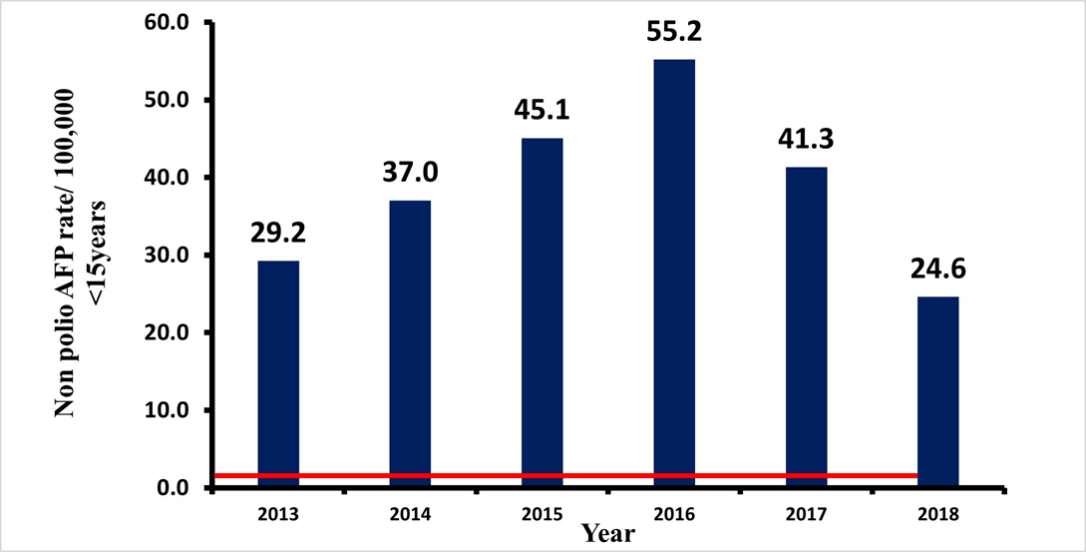
distribution of non-polio acute flaccid paralysis rate between 2013 and 2018 in Kebbi State

**Figure 4:**
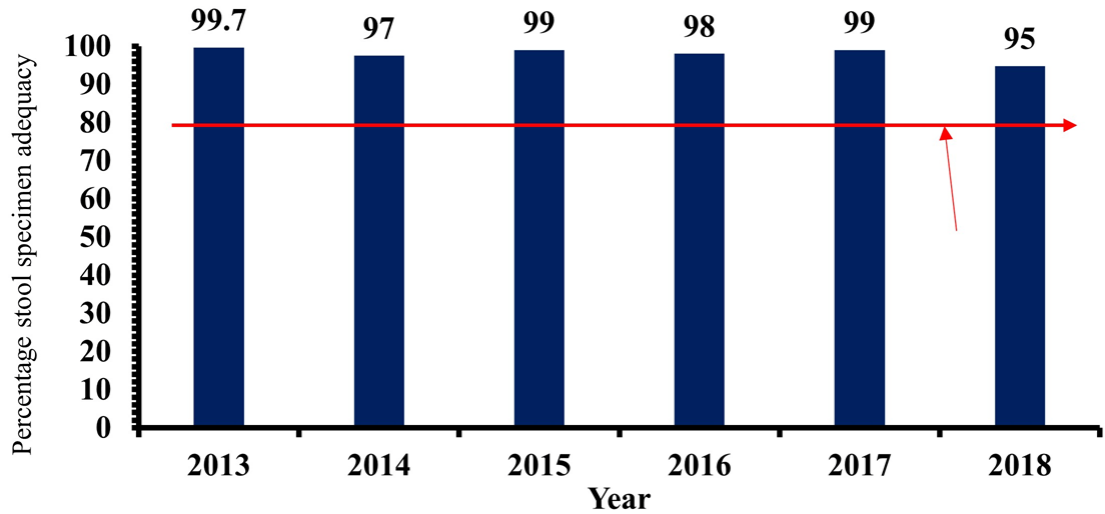
stool adequacy of acute flaccid paralysis cases reported in Kebbi State, 2013-2018

**Sensitivity of AFP surveillance system in Kebbi State, 2013-2018:** the annualized non-polio AFP rate per 100,000 children under 15 years of age, the target of >1/100,000 has been consistently achieved by the studied surveillance system throughout the review. The stool adequacy of ≥80 as prescribed by the WHO has been achieved throughout the review by the AFP surveillance system in the State. The minimum non-polio AFP rate (≥1/100,000/year of under 15) and stool adequacy rate (> 80% stool samples must be adequate) were consistently above the WHO minimum standard, as depicted in Figure 3 and Figure 4, respectively. The system is, therefore, sensitive.

**Figure 5:**
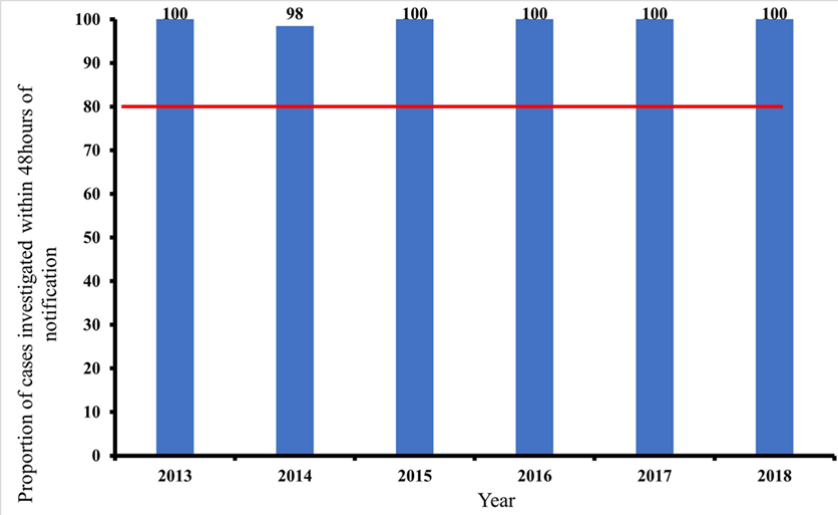
timeliness of case investigation of reported AFP cases between 2013-2018

**Figure 6:**
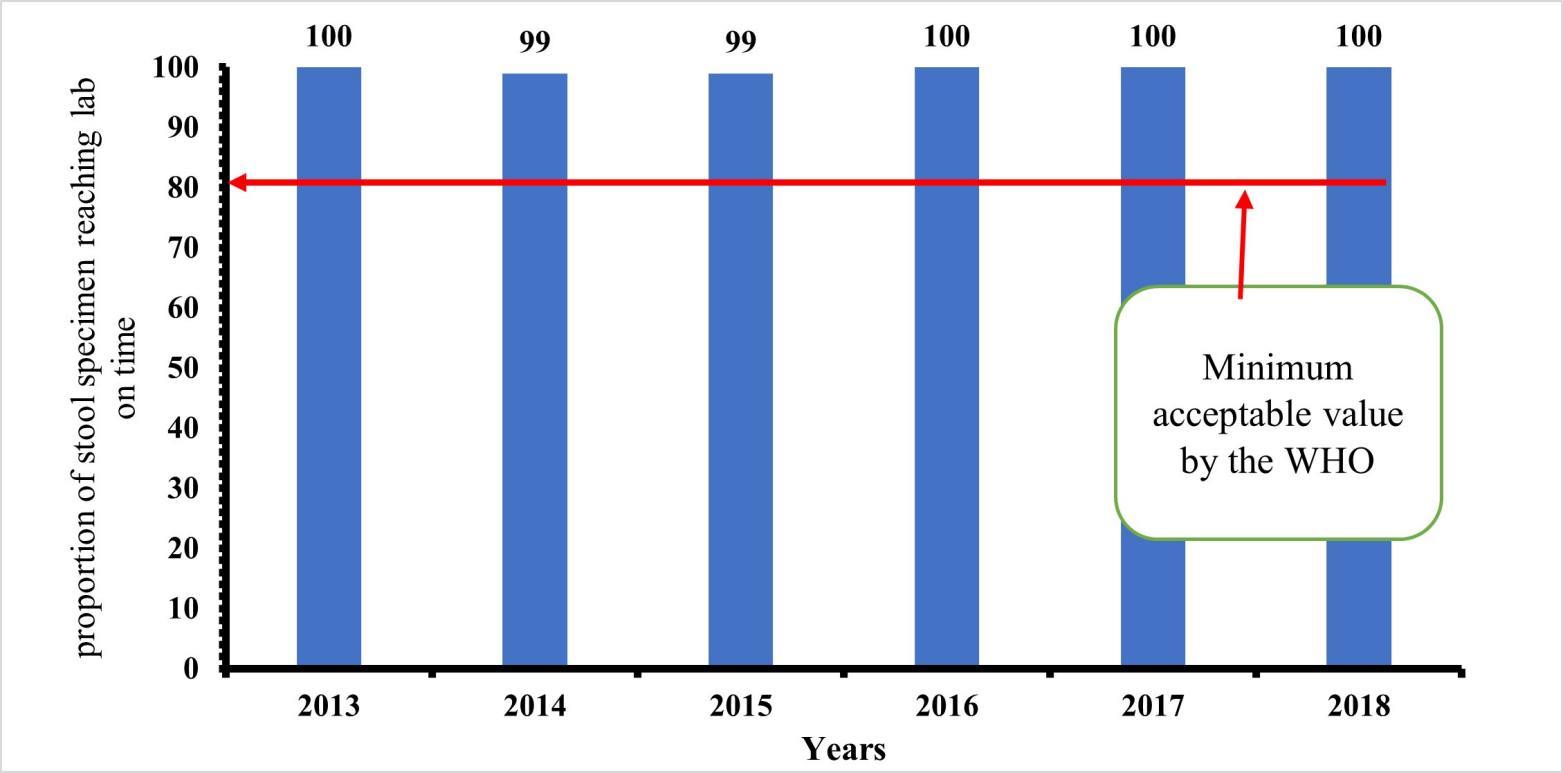
timeliness of stool sample arriving at the lab on time between 2013-2018

**Timeliness of case investigation and stool arriving lab on time:** all AFP cases should be investigated within 24-48 hours of reporting and 2 stool samples should be collected 24 hours apart, and each should be adequate. Furthermore, all collected stool samples should reach the WHO accredited laboratory in good condition and on time (48-72 hours of collection). At least 80% of cases of AFP reported, must be investigated within 24-48 hours of notification, as prescribed by the WHO. This has been consistently achieved in all the periods of review. The lowest percentage attained was 98% (2014). The minimum cut-off mark for stool samples arriving at the laboratory on time (72 hours after collection), is also 80%. This has also been achieved consistently by the AFP surveillance system in Kebbi State, Nigeria. The proportion of AFP cases investigated within 48 hours of notification as well as the proportion of stool samples arriving at the lab on time (i.e. 72 hours from being sent to the lab), between January 2013 and December 2018. Both results were consistently above the WHO minimum standard of 80%. The minimum level obtained was 98% (for timeliness of case investigation), and 99% (for timeliness of sample arriving at the laboratory on time). The stool sample must arrive at the laboratory in good condition; this simply means that the stool: must arrive at the designated laboratory on time (48-72 hours of collection) and the quantity of stool collected must be at least 8 g, not desiccated, the container and case investigation form well labelled, the container well sealed and at a good temperature of 2-8°C (reverse cold chain). Figure 5 and Figure 6 above show that the system met the minimum criteria set by the WHO and, therefore, was timely.

**Gap identified from the operational side of this study:** the private healthcare facilities in Kebbi State are not fully participating in the AFP reporting. Kebbi State does not completely own the AFP surveillance system because funding, data management, and sustainance of the DSNOs are done by donor organizations.

## Discussion

AFP surveillance system in Kebbi State was found to meet some of the attributes of a good surveillance system. The state-level surveillance officers (State Epidemiologist, State disease surveillance and notification officer) and LGA-level health workers (DSNOs) attested to the ease of operation of the system which is well structured and flexible to changes. The ease of operating the system was due to the simplicity of the data tools and case definition. These findings were similar to some studies conducted in Oyo State Nigeria [12] and another survey in Ghana [13] and Pakistan [14] where the AFP system was found to be simple and flexible. However, this is in contrast to a study in Zimbabwe, where a good number of respondents found it difficult to fill the case investigation forms- [15] and also a similar study from the same country that found poor knowledge of specimen type from the respondents [16].

The Kebbi State surveillance system is majorly donor-funded. The WHO is the major funder, which provides logistic support for transportation during active case search, transportation of samples to the designated laboratories, and provision of some laboratory reagents and consumables. The surveillance officers; mainly the DSNOs are under the State's payroll, even though their transportation and other stipends come from the WHO. This is possibly why almost all the surveillance system's attributes were achieved throughout the review period. The implication of this is, if the donors withdraw their funding today, the surveillance system may not stand on its feet. Previous studies in Oyo State, Nigeria, showed the majority of funding was also from partner agencies [12]. With this tremendous support from the WHO, the surveillance has been going on smoothly. The surveillance officers are incentivized by the donors on sample collection and transportation. However, if the donors chose to withdraw their support, the surveillance system will face a major setback.

In terms of data quality, timeliness, and completeness of reporting, the system in Kebbi State has surpassed the 80% target by the WHO, throughout the review. Similar findings were seen in Oyo State [12] but contrary to findings in another similar study from Zimbabwe wherein some cases, the reporting was not complete and not timely [16] was attributed to bad roads and lack of dedicated mobility and resources to conduct active case search and transportation of samples to the laboratory on time. We found out that the system was generally acceptable to the surveillance officers. All the respondents were willing to continue with the AFP surveillance. This is in line with similar studies [15,17]. This is probably because of incentivization and intensification of support by the WHO towards the eradication of polio. In yet another study, the AFP surveillance system was found to be less acceptable [17]. This was because about 4% of respondents felt it wasn't their duty to fill out the case investigation forms, though about 96% were willing to continue filling the forms [17]. The system was also representative. This is because the data collected were from all the tertiary and secondary health facilities across the State. From both urban and rural areas and includes all the focal primary health facilities both public and private. This is similarly found in other studies where the AFP surveillance system is representative [15,18,19]. However, in another study, the system was found not to be representative. This is because data from private clinics and hospitals were not available [17].

Our findings about the AFP surveillance system in Kebbi State are of particular importance to Nigeria and other countries in the region toward a polio eradication effort including strengthening public health surveillance systems in similar settings. Several countries in the region, just like Nigeria, have developed AFP surveillance systems as a key feature of their public health infrastructure. In the case of the global polio eradication initiatives (GPEI), the AFP surveillance works as an integral part of the initiative and has commonly recognized methodologies in terms of case detection, reporting and laboratory confirmation [20]. Another similarity in the AFP surveillance from countries in the region is the multi-stakeholder engagement that aims to align efforts of government health agencies, international organizations and local communities to coordinate and ensure effective detection and response to AFP cases [21,22]. Furthermore, many countries depend on donor funding and technical support to maintain their AFP surveillance systems [23]. However, this reliance emphasizes the need for sustainable funding mechanisms to support and scale these efforts in the long term.

Despite pattern similarities in patterns, countries differ in resource allocation, health system capacity, and technology adoption. While some, have advanced surveillance systems, others struggle due to limited resources. Health system capacity also varies with disease burden and infrastructure, affecting AFP surveillance effectiveness [24,25]. These similarities and differences are essential to understanding when to strengthen AFP surveillance in the region. Countries can strengthen their surveillance and improve their response toward AFP and other public health threats by exchanging best practices, lessons learnt, and innovative solutions. This information can guide policymakers and public health officials to develop country-specific strategies, thereby contributing to the global goal of polio eradication and improved health outcomes [26,27].

Throughout the review (2013-2018), the AFP surveillance system in Kebbi State was found to be sensitive. The non-polio AFP rate and the stool adequacy were consistently above the WHO minimum standards. Similar results were found in other surveys [14,28]. In other studies, the AFP surveillance system was found not to be sensitive [15,16]. An uninvestigated case was found in a hospital in one of the studies [29], and in another study, the insensitivity of the system was due to a failure to report a case as a result of a lack of resources to conduct active case search, and transport stool specimens on time [16].

**Limitations:** the study only included data from a certain subset of health facilities in Kebbi State which may affect generalizability; health facilities were more likely to report on cases of AFP that were severe and the study only looked at data for six years (from 2013 to 2018). This may not be enough time to capture long-term trends in AFP surveillance.

## Conclusion

The performance of the AFP surveillance system in Kebbi State, Nigeria, was excellent. Most of the major stakeholders found the surveillance system to be useful and acceptable as it was able to detect cases. The surveillance system also played an important role in assessing the effectiveness of the current AFP control strategies. However, the system was discovered to be highly donor-dependent for funding and technical support. Private healthcare facilities currently not reporting should be encouraged to report. Finally, Kebbi State should take complete ownership of the AFP surveillance system and ensure its sustainability by providing funding and logistic support.

### What is known about this topic

- AFP surveillance is a vital part of polio eradication efforts, it helps to detect and interrupt polio transmission by monitoring children under 15 years of age with sudden limb weakness;
- An effective AFP surveillance system is necessary for verifying the absence of wild poliovirus in polio-free and polio endemic regions;
- Key performance indicators such as non-polio AFP rates ≥2 per 100,000 children under 15 years and stool adequacy rates ≥80% are globally recognized benchmarks for evaluating the sensitivity and quality of AFP surveillance systems.

### What this study adds

- The AFP surveillance system in Kebbi State is well-integrated with the Integrated Disease Surveillance and Response (IDSR) system;
- There is a need for regular training and retraining of surveillance officers for the optimum performance of the AFP surveillance system;
- Private healthcare facilities are not fully reporting suspected AFP cases, and their presence can be used to strengthen the system.
